# Analysis of individual-level data from 2018–2020 Ebola outbreak in Democratic Republic of the Congo

**DOI:** 10.1038/s41598-022-09564-4

**Published:** 2022-04-01

**Authors:** Harley Vossler, Pierre Akilimali, Yuhan Pan, Wasiur R. KhudaBukhsh, Eben Kenah, Grzegorz A. Rempała

**Affiliations:** 1 College of Public Health, The Ohio State University, Columbus, OH USA; 2 College of Public Health, University of Kinshasa, Kinshasa, Congo; 3 School of Mathematical Sciences, University of Nottingham, Nottingham, UK

**Keywords:** Computational biology and bioinformatics, Computational models, Ecological epidemiology, Mathematics and computing

## Abstract

The 2018–2020 Ebola virus disease epidemic in Democratic Republic of the Congo (DRC) resulted in 3481 cases (probable and confirmed) and 2299 deaths. In this paper, we use a novel statistical method to analyze the individual-level incidence and hospitalization data on DRC Ebola victims. Our analysis suggests that an increase in the rate of quarantine and isolation that has shortened the infectiousness period by approximately one day during the epidemic’s third and final wave was likely responsible for the eventual containment of the outbreak. The analysis further reveals that the total effective population size or the average number of individuals at risk for the disease exposure in three epidemic waves over the period of 24 months was around 16,000–a much smaller number than previously estimated and likely an evidence of at least partial protection of the population at risk through ring vaccination and contact tracing as well as adherence to strict quarantine and isolation policies.

## Introduction

We present here a quantitative analysis of the effects of public health interventions against the spread of the Ebola virus disese (EVD) during the DRC Ebola epidemic that unfolded between August 2018 and September 2020 in the northeastern provinces of DRC^1,2^, partially sharing the timeline of the better known and much larger West African epidemic^3^. The DRC 2018 epidemic, being more geographically contained and smaller, was considerably better documented, with the majority of cases’ disease histories collected through the efforts of the College of Public Health at the University of Kinshasa^4^. The work of these researchers allowed in particular for tracking the time elapsed between symptom onset, hospitalization, and recovery or death for over 3000 Ebola victims, creating a unique opportunity for detailed analysis of the epidemic dynamics based on individual disease histories.

The authorization for emergency use of Merck experimental Ebola vaccine rVSV-ZEBOV-GP^5,6^ and its field deployment in 2019 has provided for better protection of those involved in monitoring efforts, as it was given to many frontline workers including doctors, nurses, and burial workers. An estimated 330,000 people living in the northern DRC provinces were vaccinated in 2019 and 2020, including frontline workers as well as ring vaccinations of the contacts of suspected and confirmed cases. This was done in part by the international nongovernmental organization Doctors Without Borders, with authorization by the Ministry of Health, concerned with the possibility of further northward spreading of the disease^1^. However, the more comprehensive vaccination efforts were complicated and significantly delayed in late 2019 and in 2020 by local distrust, political instability and the resulting lack of security both for aid workers and for vaccine supplies^7^. For those reasons, despite the apparent effectiveness of the rVSV-ZEBOV-GP Ebola vaccine, quarantine and isolation were often still the primary and most effective practical interventions for breaking the chain of transmission, especially in rural and isolated communities across northern DRC.

Early in the outbreak, a large number of health care workers working for the DRC ministry of health were brought to the villages to monitor possible EVD symptoms as the ring vaccination campaign was introduced wherever adequate vaccine supplies were procured and safe funeral practices were mandated^8^. All these factors likely limited the size of the initial outbreak and prevented the uncontrolled EVD spread into the crucial commercial centers of the region along the border towns of Goma in North Kivu and Gisenyi in Rwanda^9^. The spillover of DRC cases to Rwanda and possibly Uganda would have undoubtedly and considerably increased the geographical reach of the outbreak. Largely due to successful public health monitoring efforts, EVD spread occurred mainly via symptomatic individuals in relatively isolated villages, which contributed to better protection of neighbors and other household members of EVD victims and the lack of transmission in the treatment centers and among health care workers. This simplified transmission chain allowed us, in turn, to implement a relatively simple mathematical model of infection spread based on an individual-level stochastic SIR (susceptible-infected-recovered) model^10^.

The classical SIR model for epidemic dynamics was introduced in early 20th century for malaria and cholera and led to the so-called ecological models of infections usually described by ordinary differential equations (ODEs)^11^. Such models typically represent an epidemic as a process of transferring individuals between disease-related states (or compartments) and describe it in terms of the temporal changes in the compartment sizes. For the purpose of our analysis, we consider a version of that classical model, which focuses on the fate of a single individual (or agent), making our approach similar to the modern agent-based model (ABM) approach to disease modeling^12^. Although other more complex ecological models have been used for studying Ebola transmission (most notably including “funeral” and “exposed” compartments, see^13^), it appears that for 2018 DRC Ebola data our stochastic SIR model is both sufficiently flexible to incorporate the heterogeneity of individual disease histories and simple enough to require only a small set of population-level parameters. This allows us to estimate the key quantities of interest in the DRC outbreak, such as the rates of disease reproduction and quarantine/isolation (or hospitalization) and the size of the subpopulation at risk of infection though contact with EVD cases. The model also accounts for observed seasonality and spatial variation in the number of cases (e.g., see^14^) by allowing for the three independent sets of parameters to govern the three waves of infections observed over the course of the outbreak. For the purpose of our analysis we have determined, similarly as in^7^, the first wave to end in late February 2019 and the second one to end around late May 2020. See Table 1 below for more details. Our approach may be also viewed as an alternative to the complicated multi-phase longitudinal analysis proposed recently for the DRC outbreak data in^15^.

## Materials and methods

### Ebola dataset

The 2018–2020 DRC EVD outbreak lasted over 24 months and spread over 3 distinct spatial and temporal waves. Between the emergency declaration of the EVD outbreak in northern DRC on August 1, 2018 and the outbreak’s official end on June 25, 2020, the DRC Ministry of Health has reported a total of 3481 cases (including confirmed and probable), 1162 recoveries, and 2299 deaths^16^ in the provinces of Northern Kivu, Southern Kivu, and Ituri. The dataset considered here is a large subset of the entire EVD database compiled by the University of Kinshasa School of Public Health, which comprises 3117 total case records (confirmed and probable) recorded between May 3, 2018, and September 12, 2019. The data included partially de-identified but still detailed patient information, such as each person’s location, date of symptom onset and hospitalization, as well as discharge due to recovery or death. These individual records came from the Ebola treatment centers in 24 different health zones, spread out among the three DRC provinces of Northern Kivu, Southern Kivu, and Ituri.

**Figure 1:**
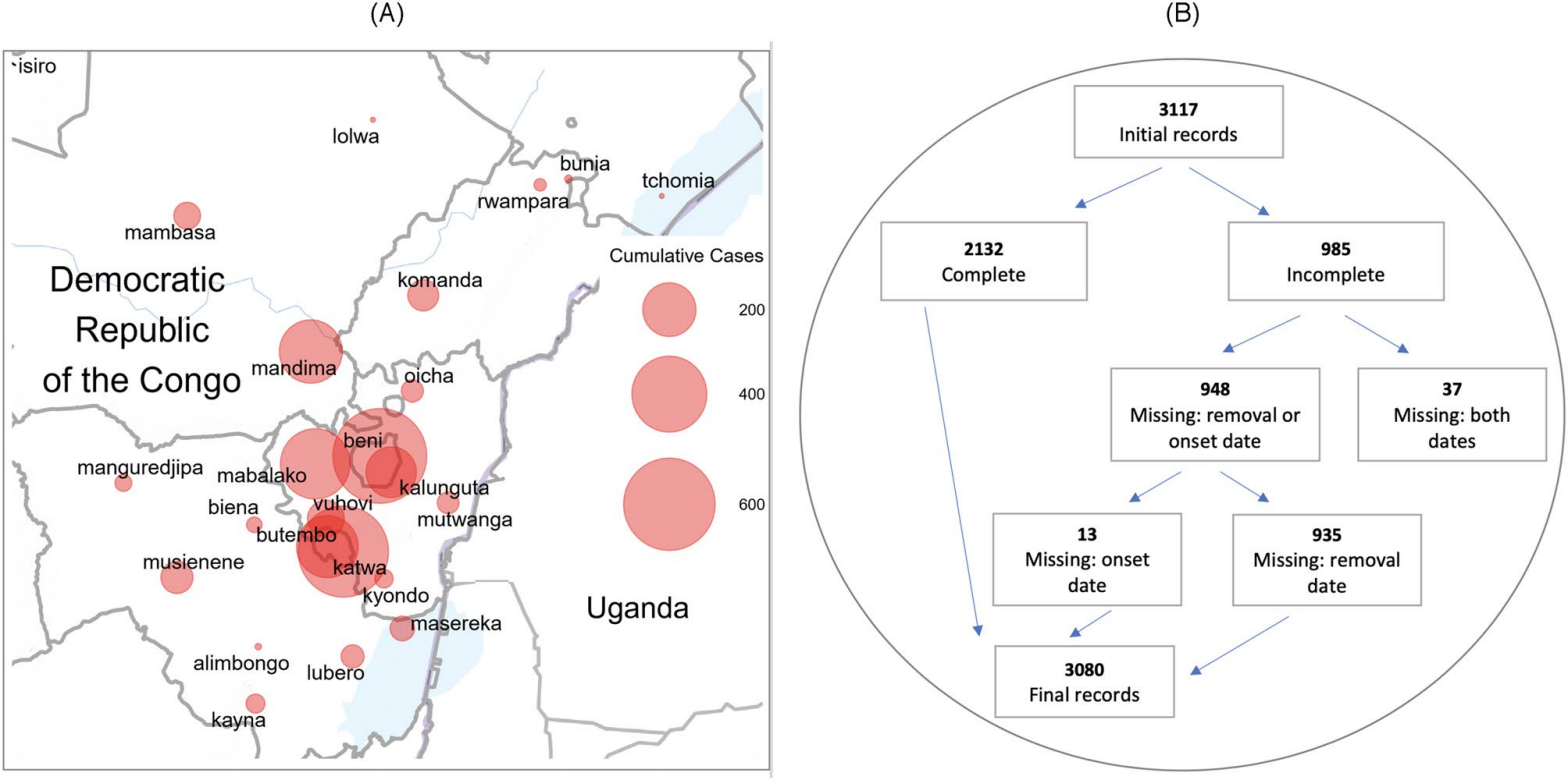
DRC Ebola dataset. (**A**) The spatial distribution of 3481 EVD cases across the northern DRC health zones during Ebola 2018–2020 outbreak. (**B**) The flowchart of personal records available up to September 12, 2019 available for the current analysis. The total number of available individual disease records was 3080. Map created using open software R^17^ with geospatial data obtained from^18^.

Of the 24 health zones, 77.1% of all cases were from only 6: Beni, Butembo, Katwa, Kalunguta, Mabalako, and Mandima. Only 9.7% of cases were under the age of 18. There is also a slightly larger proportion of females contracting the disease, comprising 57.0% of the cases. Approximately 5% of the cases were health care workers. About one-third of the EVD fatalities were not identified until patient’s death and thus not effectively isolated from the time of infection. Although over 170,000 contacts of confirmed and probable Ebola cases had been monitored across all affected health zones for 21 days after their last known exposure by the end of the epidemic, some of the contact tracing was incomplete due to insecurity that prevented public health response teams from entering some communities. The overall case density map is presented in panel (A) of Fig. 1 with the animated version of the map presented in the online appendix in Fig. A.1. Notice that the high-density areas, particularly Butembo, Katwa, and Beni, are all spatially small health zones corresponding to cities or towns with larger populations.

**Figure 2:**
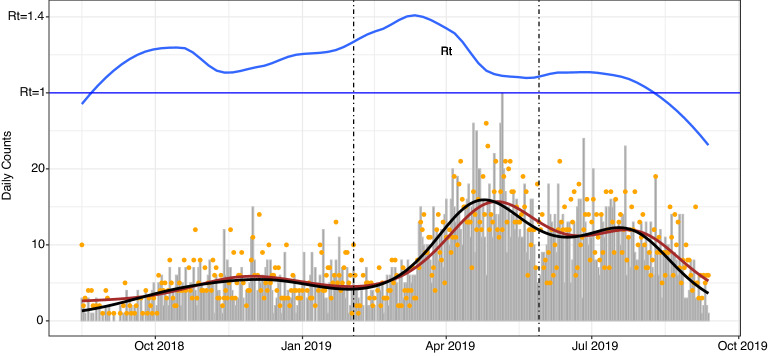
Daily incidence and removal rates. Daily incidence (grey bars) and removal counts (red dots) during DRC Ebola 2018–2020 outbreak between August 15, 2018 and September 12, 2020 along with their respective trendlines (loess smoothers). The blue trendline above the plot represents daily effective reproduction number $\mathcal{R}_t$ defined as the ratio of daily number of new infections to new removals. The vertical lines indicate cut-off dates for data collection in each wave as listed in Table 1.

**Table 1:** Observed cases by EVD wave.

	Wave 1	Wave 2	Wave 3	Total
Cut-off dates	February 27, 2019	May 27, 2019	September 12, 2019	
No. cases	907	1104	1477	3481
Most affected Health zones	Beni, Katwa	Butembo, Katwa, Mabalako, Mandima	Beni, Kalunguta Mandima	

The observed cases aggregated by 3 infection waves and the corresponding cutoff date for data collection. The number of cases observed in wave 3 by September 12 was 1113 with a combined total of 3117 cases across all three waves (see Fig. 1).

#### Case alerts and definitions

Since early August, 2018, the DRC Ministry of Health has been collaborating with several international partners to support and enhance EVD response activities through its emergency operations center in Goma. To the extent possible given regional security considerations^19^, the response teams were deployed to interview patients and their suspected contacts using a standardized case investigation form classifying cases as suspected, probable, or confirmed. A suspected case (whether surviving or not) was defined as one with the acute onset of fever (over 100$^{\circ }$F) and at least three Ebola-compatible clinical signs or symptoms (headache, vomiting, anorexia, diarrhea, lethargy, stomach pain, muscle or joint aches, difficulty swallowing or breathing, hiccups, unexplained bleeding, or any sudden, unexplained death) in a North Kivu, South Kivu, or Ituri resident or any person who had traveled to these provinces during this period and reported the signs or symptoms defined above. A patient who met the suspected case definition and died but from whom no specimens were available was considered a probable case. A confirmed Ebola case was defined as a suspected case with at least one positive test for Ebola virus using reverse transcription polymerase chain reaction (RT-PCR)^20^ testing. Patients with suspected Ebola were isolated and transported to an Ebola treatment center for confirmatory testing and treatment^2^.

#### Onset and removal

In our analysis of the DRC dataset, we focused on dates of symptom onset and removal, with removal defined as either a death/recovery at home or transfer to an Ebola treatment center (ETC). It was assumed that, once in the treatment center, the probability of further infection spread by an isolated individual was very small due to the strict safety protocols—and later due also to vaccination of healthcare personnel and family members who were in contact with the suspected Ebola case. As summarized in panel (B) of Fig. 1, we were able to access 3117 out of 3481 individual records of confirmed and probable Ebola cases. Of these 3117 records, 37 were missing both the onset and recovery dates and were removed from further analysis. In about 30% of the remaining records, either their dates of onset or removal were missing. A detailed flow diagram summarizing the amount of missing data and data processing leading to the final dataset is presented in panel (B) of Fig. 1. The distribution of the original and the partially imputed records across the three waves of infection is provided for further reference in Table 1.

#### Spatial and temporal patterns

Throughout the pandemic, the incidence rates exhibited strong spatial and temporal patterns that can be summarized as three distinct waves of infections with approximate boundaries marked by vertical lines in Fig. 1. The distribution of weekly reported cases across the most affected health zones listed in Table 1 is provided in the bar plot and in the corresponding animation in the appendix (see Figure A.1). As seen from the bar chart and the animated plot, the epidemic was initially driven largely by infections in the health zones of Beni, Mandima and Mabalako. After several months, the incidence of new cases in these zones subsided, but the epidemic moved south to the health zones of Katwa and Butembo, where the majority of new infections was registered between weeks 22 to 45 of the epidemic (see Panel (A) in Figure A.1 in the online Appendix). In the final spatial shift, around week 49, the epidemic returned to the health zones of Beni, Mandima, and Mabalako, where it was mostly extinguished around week 60 (September 2019). Isolated Ebola incidences occurred sporadically across northern DRC until end of the outbreak was officially declared in June 2020.

The empirical patterns of incidence and removal for EVD cases are summarized in Fig. 2 with the bar and the dot plots representing the daily numbers of new infections and removals, respectively. As seen from the plot, these daily counts closely follow a three-wave temporal pattern in Table 1. This is further evident from the black and red trendlines representing the loess smoothers (see^21^). The daily ratio of new cases and removals may be interpreted as a crude estimate of the *effective reproduction number* $\mathcal{R}_t$ defined more formally in (2) in Model for Data Analysis below. In particular, the blue trendline for $\mathcal{R}_t$ indicates that towards the end of the observed time period, the number of removals outpaced the number of new infections ($\mathcal{R}_t <1$). The ability to sustain this pattern for a sufficiently long time period, mostly by increasing the rate of quarantine and ETC transfers along with ring vaccination of case contacts was largely credited with the end of EVD epidemic in mid-2020. The quantification of this public health intervention effect in 2018–2020 DRC outbreak is one of the main motivations for our model-based analysis. Although the precise cut-off dates for the three waves of 2018–2020 Ebola infections are difficult to establish, the incidence data along with simple statistical analysis (see Parameter estimation) indicate that the first wave lasted approximately until the end of February 2019, whereas the second wave ended around the end of May 2019. For the purpose of the data analysis below, the specific break dates used were February 27, 2019 and May 27, 2019 as marked by vertical lines in Fig. 2. September 12, 2019 was the cutoff date for the individual records data available from the University of Kinshasa (see Table 1).

### Model for data analysis

The analysis of the individual-level epidemic data is based on the standard ecological model known as the SIR (susceptible-infected-removed) model and developed for the purpose of analyzing average behavior of a large population with a homogenous pattern of interactions^11,22^. Although there are many variants of SIR models in the literature^23^, our current analysis considers the classical Kermack-McKendrick SIR model that assumes the proportions of population categorized as susceptibles (*s*), infected ($\iota$), or removed (*r*) to evolve according to the differential equations

1 $$\begin{aligned} \begin{aligned} {\dot{s}}_t&= -\beta s_t \iota _t , \\ {\dot{\iota }}_t&= \beta s_t\iota _t - \gamma \iota _t , \\ {\dot{r}}_t&= \gamma \iota _t, \end{aligned} \end{aligned}$$

with $s_0 = 1, \iota _0 = \rho >0$ and $r_t = 0$ where $\beta > 0$ is the rate of infection, $\gamma > 0$ is the rate of recovery and $\rho > 0$ is the initial amount of infection. In particular, the model implies the existence of the basic reproduction number $\mathcal{R}_0$ (R-naught), which determines the average speed of disease spread^11^ and is given by the formula

$$\mathcal{R}_0=\beta /\gamma .$$

If $\mathcal{R}_0 > 1$, the proportion of infected initially rises and then subsides, with the final proposition of surviving susceptibles given by $s_\infty = 1 - \tau > 0$ where $\tau$ is know as the epidemic’s *final size*. In typical statistical analysis, an estimate of $\mathcal{R}_0$ is obtained by separately estimating the parameters $\beta$ and $\gamma$. Another important quantity related to (1) is the *effective reproduction number*, which is typically defined as

2 $$\begin{aligned} \mathcal{R}_t= \mathcal{R}_0 s_t. \end{aligned}$$

Although equation (1) is typically considered in the context of an average behavior of a large population, for our purposes we interpret it as defining the individual histories of infection and recovery, according to the idea of the dynamic survival analysis (DSA) discussed recently in^10^ and^24^ and also briefly summarized in the Appendix. With the DSA approach, we interpret equation (1) as the so-called stochastic *master equation*^25^ describing the change in probability of a randomly selected individual being at time *t* either susceptible, infected, or removed. These respective probabilities are represented by the scaled proportions $s_t/(1+\rho )$, $\iota _t/(1+\rho )$, and $r_t/(1+\rho )$ and evolve according to (1). As outlined in^10^, the DSA-based interpretation of the classical SIR equations has a number of advantages that make it particularly convenient for analyzing epidemic data consisting of individual histories of infection onsets and removals, which is exactly the type of data available in the DRC Ebola dataset. The fact that the model is individual-based implies also that we can vary the parameters $\theta =(\beta ,\gamma ,\rho )$ to account for individual covariates and changes in the parameter values over time, as different waves of infection sweep through the population. Finally, for the purpose of our analysis, it is also important to note that the DSA model does not require any knowledge of the size of the susceptible population subjected to the epidemic pressure. For the DRC dataset, that assumption would be difficult to justify due to spatial and temporal heterogeneity of the epidemic and the frequent movements of local populations driven by political conflicts and insecurity. Another element complicating the determination of the size of susceptible population was the ring vaccination campaign that has been conducted since 2019 wherever possible in the northern DRC during periods of relative stability, despite local mistrust and supply issues. This campaign ultimately resulted in over 250,000 vaccinations.

Note that, because $s_0 = 1$, the values of $\mathcal{R}_0$ and $\mathcal{R}_t$ coincide for $t = 0$. Moreover, $s_t = \exp \left( -\mathcal{R}_0 \int _0^t r_u \mathrm {d}u \right)$ is a decreasing function of time and therefore, so is $\mathcal{R}_t$. However, in practice, this implication is problematic. Rewriting $\mathcal{R}_t = - {\dot{s}}_t/ {\dot{r}}_t$ suggests that a crude but sensible way to estimate $\mathcal{R}_t$ empirically is to take the ratio of daily number of new infections to new removals. The empirical $\mathcal{R}_t$ thus estimated will not be necessarily monotonically decreasing. In the light of possibly changing parameters and the effective population size, we have adopted this approach to estimating the daily effective reproduction number $\mathcal{R}_t$ in Fig. 2.

### Parameter estimation

We assume that, for each of the three waves of the epidemic, we have a separate and independent set of parameters $\theta$ and that, in each wave, we observe $n_T$ histories (records) of infection. The *i*-th individual history may be represented either by the times of disease onset and removal $(t_i,T_i)$ or by $t_i$ or $T_i$ times alone $(t_i,\circ )$ or $(\circ ,T_i)$ ($\circ$ denoting missing value). We assume that among the available $n_T$ histories we have *n* complete records $(t_i,T_i)$, $n_1$ incomplete ones $(t_i,\circ )$ and $n_2$ incomplete ones $(\circ ,T_i )$. The wave-specific DSA likelihood function for *n* complete data records is (see Appendix)

3 $$\begin{aligned} \begin{aligned} {\mathcal {L}}_C(\theta \vert t_1\ldots ,t_n,T_1,\ldots ,T_n,T)=(s_T-1)^{-n}\prod _{i=1}^n {\dot{s}}_{t_i}\gamma ^{w_i}e^{-\gamma (T_i \wedge T -t_i)} \end{aligned} \end{aligned}$$

where *T* is the available time horizon and $w_i$ is the binary variable indicating whether $T_i$ is right-censored (that is, $T_i\wedge T =T$) in which case $w_i = 0$ and otherwise $w_i = 1$. For the remaining $n_1+n_2$ records that are partially incomplete, the wave-specific DSA likelihood function is

4 $$\begin{aligned} \begin{aligned} {\mathcal {L}}_I(\theta \vert t_1\ldots ,t_{n_1},T_1,\ldots ,T_{n_2},T)= (s_T-1)^{-(n_1+n_2)} \gamma ^{n_2}\prod _{i=1}^{n_1} {\dot{s}}_{t_i} \prod _{i=1}^{n_2} (\rho e^{-\gamma T_i }-\iota _{T_i}) \end{aligned} \end{aligned}$$

where we assume that $T_i<T$. The overall likelihood for all $n_T$ individual histories is obtained by multiplying (3) and (4). Note that the likelihood formulas depends on the parameter $\beta$ only implicitly, through the values of the function $s_t$ defined by (1). Note also that we assume *T* to be unique and exactly known although in practice this may not be true as subsequent waves of infection may be too close in time (perhaps even overlapping) to allow for a precise specification of *T*. In our analysis below, we solve this practical problem by considering several candidates for the values of *T* in each wave and then identifying ones that jointly maximize the combined posterior distribution corresponding to the wave-specific likelihoods in equations (3–4).

The fitting of the model parameters $\theta =(\beta ,\gamma , \rho )$ by maximizing the likelihood function (3) can be conveniently integrated into the Bayesian estimation framework, which allows for a more complete propagation of uncertainty and the use of external information in the statistical model. This, in turn, allows us to produce estimates that reflect all available information and uncertainty. In our DRC data analysis, the approximate posterior densities of $\theta$ were obtained using the Hamiltonian Monte-Carlo sampler^26^ implemented in the open source statistical software STAN^27^ and integrated with the popular statistical analysis language R via the library Rstan^28^. For the Rstan analysis, we have assumed uniform (sometimes improper) prior distributions on the $\theta$ components as follows

5 $$\begin{aligned} &\beta \in (0.15, \infty ), \\&\gamma \in (0, \beta ),\\&\rho \in (0, 1). \end{aligned}$$

The lower bound was placed on $\beta$ based on empirical information, and the upper bound was placed on $\gamma$ to enforce the constraint $\mathcal{R}_0>1$. Given the wave-specific time horizons (*T*’s), the set of parameters for each epidemic wave was estimated independently using 2 independent chains of 3000 iterations, with a burn-in period of 1000 iterations. The chains’ convergence assessed using Rubin’s R statistic^28^. The analysis resulted in approximate samples from the posterior distribution of $\theta$ for each of the three waves of the epidemic (see e.g., Fig. 4).

#### Ethics statement on human subjects and methods

The research was conducted in accordance with the relevant guidelines and regulations of the US law and OSU Institutional Review Board. The research activities involving human subjects discussed in the paper meet the US federal exemption criteria under 45 CFR 46 and 21 CFR 56.

## Significance statement

With the world health community largely preoccupied with the current COVID-19 pandemic, the Ebola Virus Disease (EVD) continues to lurk as a significant threat to public health, prosperity, and political stability in large regions of Africa with undiminished potential for spread to other parts of the world. Despite its vital importance for public health policy, knowledge about the effects of the recent 2018–2020 EVD response efforts in the Democratic Republic of the Congo (DRC) based on ring vaccination supplemented with isolation and quarantine has been limited by challenges with data collection and by the lack of simple methods for analyzing complex multi-wave patterns of disease incidence occurring across time and space. Within this environment, competing narratives with differing policy implications emerged around the effectiveness of vaccination strategy and the need for supporting DRC Ebola treatment centers. To address this issue, University of Kinshasa researchers collected a large number of individual records of disease histories from probable and confirmed Ebola cases during the 2018–2020 EVD outbreak in DRC. This study describes a model-based Bayesian statistical method developed to estimate the effects of ring vaccination, quarantine, and isolation in Ebola treatment centers across northwestern provinces of the country. The method accounts in particular for missing and censored data, heterogeneity of infection patterns and multiple waves of infection with different intervention strategies.

## Results

**Figure 3:**
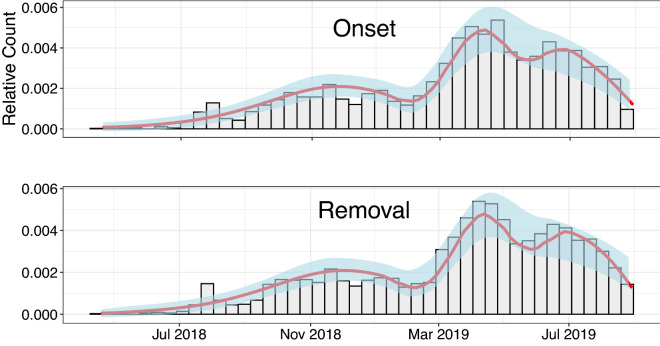
Model fit. Comparison of the statistical model fit (red curve) with the observed daily incidence (onset) and removal relative counts for all 3 waves of the epidemic combined. The shaded region indicates the 95% credibility bounds based on the posterior distributions of the model parameters estimated separately for each wave with values summarized in Table 2.

The overall comparison of the parametric DSA model predictions with the empirical data in DRC dataset until September 12, 2019 is given in Fig. 3, where the scaled theoretical densities of the epidemic are plotted alongside the observed relative daily counts of infection (onset) and removal shown earlier in Fig. 2. As seen from the plots, the multi-wave model appears to capture well the empirically observed patterns of daily counts represented by the histogram bars. The 95% credibility bounds around the model fit (marked in blue) are calculated based on the model parameter posterior distributions estimated via the MCMC algorithm with priors described in Parameter estimation. We note that, although the DSA fit curve appears quite similar to the non-parametric loess smoother presented in Fig. 2, the parametric fit has an advantage of providing an explicitly interpretable set of parameters describing the outbreak dynamics. This allows, for instance, for a purely quantitative comparison of the 3 different epidemic waves.

**Table 2:** Parameter estimates.

Parameter	Wave 1	Wave 2	Wave 3
*T*	300 days	89 days	108 days
$\beta$	0.190 (0.178, 0.204)	0.217 (0.201, 0.232)	0.235 (0.218, 0.253)
$\gamma$	0.169 (0.157, 0.183)	0.179 (0.165, 0.192)	0.214 (0.199, 0.230)
$\rho$	0.00021 (0.00016, 0.00027)	0.0054 (0.0044, 0.0065)	0.0067 (0.0055, 0.0081)
$\mathcal{R}_0$	1.124 (1.108, 1.142)	1.214 (1.168, 1.262)	1.098 (1.061,1.135 )
	All Waves		
${\hat{K}}_\infty$		3481.41 (2877.416, 4155.878)	
${\hat{N}}$		16385.61 (14416.33, 18703.71)	

Wave-specific posterior estimates (means and 95% credibility bounds) from the parametric model in equation (3).

**Figure 4:**
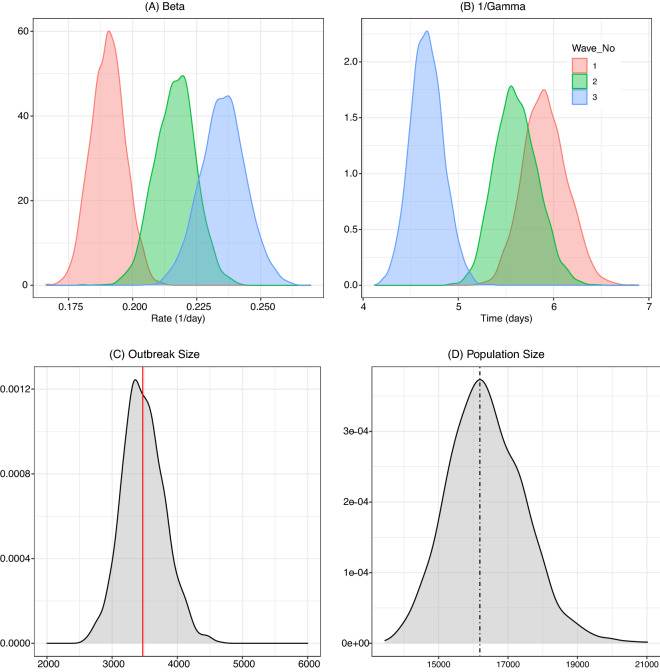
Top panels: parameters in different waves. The posterior distributions of $\beta$ and $1/\gamma$ parameters for each of the three epidemic waves. The large increase in the rate of infection between waves 1 and 2 is clearly visible in panel (**A**). In panel (**B**), the density of $1/\gamma$ represents the distribution of time from symptom onset to removal. Between wave 1 and wave 3 of the epidemic, the average time shortened from 6 to 4.6 days. Bottom panels: outbreak size and effective population size. (**C**) The posterior density of the outbreak size based on the statistical model and the actual number of observed EVD cases (vertical red line). (**D**) The posterior density of the effective population size for the epidemic. The vertical line corresponds to the empirical outbreak size (red line in panel (**C**)).

The wave-specific results of the MCMC analysis are summarized in Table 2 with some of the posterior plots presented in Fig. 4. In Table 2, the posterior mean and corresponding credibility interval for each component of $\theta =(\beta ,\gamma ,\rho )$ are listed for each epidemic wave along with the estimated reproduction numbers. Additionally, in the last two rows, the posterior estimates of the effective population size (*N*) and the outbreak size ($K_\infty$) are reported (see Appendix Section B for formal descriptions of these quantities). The MCMC estimation scheme that produced the numerical values listed in the table was based on the wave-specific likelihood functions in equation (3) conditioned on the observation periods (*T*) according to the cut-off dates in Table 1. As seen from the entries of Table 2 and from the posterior density plots in Fig. 4, the parameter values for the infection rate $(\beta )$, recovery rate ($\gamma$) and the initial prevalence of infection $\rho$ all differ considerably across waves. The most notable appears to be an average increase of 14% in the posterior $\beta$ values between waves 1 and 2. This change is seen to correspond to an 8% increase in the value of the posterior mean of $\mathcal{R}_0$ and the subsequent increase in the number of infections in wave 2 of the EVD outbreak. Another interesting observation in Table 2 is that, while the average value of $\beta$ increased moderately (about 8%) between epidemic waves 2 and 3, the corresponding average value of $\gamma$ increased over twice as much (almost 20%). Recalling the plot of the empirical effective reproduction number in the top part of Fig. 2, it appears that this increase was crucial in ultimately controlling epidemic growth and ending the outbreak within the next several months. We note that the increase in the removal rate $\gamma$ corresponds to the decrease in the duration of the infectious period $1/\gamma$ (measured in days). Thus, the increase in the respective $\gamma$ values corresponds in this case to a decrease in the average infectious period from 5.6 days to 4.7 days. This could be further compared with the average infectious period in the initial wave 1 of the epidemic, which was estimated by the model at almost 6 days (corresponding to the posterior mean $\gamma =0.169$). These differences in the wave-specific estimates of $\beta$ and $1/\gamma$ are also clearly seen in their posterior density plots in the top panels of Fig. 4, and they appear to be consistent with the empirical onset and removal rates shown in Fig. 2.

As already indicated, one of the advantages of the parametric DSA approach is that it does not require knowledge of the underlying susceptible population size but may instead infer that value from the incidence data and the estimated epidemic parameters (see Appendix Section B). The posterior means and 95% credibility bounds for the outbreak size ($K_\infty$) and the effective population size (*N*) are listed in the last two rows of Table 2, and their posterior densities are presented in the bottom panels of Fig. 4. In the bottom-left panel (C), we compare the model-predicted size of an outbreak (represented by posterior density contour with the mean of 3481.4) to the number of cases officially reported by DRC health officials at the end of the epidemic in June 2020 (represented by the red vertical line at 3,481). The effective population size corresponding to that value is marked by the vertical line in the posterior density plot in the bottom-right panel (D). Both vertical lines appear close to the modes of the posterior distributions indicating good agreement of the model-based estimates with empirical data. Note that the model predicted effective population size corresponding to the observed outbreak size is only around 16,000 with the posterior CI between 14,416.33 and 18,703.71, which is a much smaller number than one might expect based on demographic estimates (see also Conclusions).

## Conclusions

Outbreaks of Ebola in Africa are a persistent threat not only to global public health but also to economic and political stability in some of the world’s poorest and most vulnerable regions. Despite early evidence of effectiveness of the ring vaccination effort, the prolonged political and armed conflict in northern DRC, where the latest public health intervention took place, has seeded mistrust towards local authorities and international partners. This has impeded effective community collaboration, complicating the vaccination campaign and the overall response strategy^19^. To evaluate the effects of public health response to EVD outbreak in DRC during 2018–2020, we used the individual-level data based on case ascertainment, vaccination records, and contact enumeration collected by researchers at the University of Kinshasa School of Public Health in collaboration with local health authorities in northern DRC from August, 2018 to September, 2019. The analysis of this dataset is crucial for informing current and future EVD intervention policies and strategies regarding vaccination, quarantine, and isolation. However, the analysis is also quite challenging due to incomplete or missing patient information as some families have resisted putting their loved ones in isolation and some individuals have absconded from Ebola treatment centers. Another challenge is the complexity of the data itself, with individual patient histories spanning multiple waves of infections across multiple seasons and spatial environments result in very heterogenous and sometimes incompatible health records.

To overcome these challenges and analyze the University of Kinshasa dataset, we employed the dynamic survival analysis (DSA) method^10^, which combines an individual-level Bayesian survival model with a classical SIR epidemic modeling framework. The fusion of the two allowed us to coherently integrate multiple analyses of individual disease histories into a single analysis based on a simple parametric model. Using that model, we were able to estimate the reproduction numbers and the effective population sizes in each of the three major waves of the EVD epidemic while appropriately accounting for uncertainty due to heterogeneity, missingness, or censoring in the records of EVD patients. This Bayesian framework also allowed us to incorporate external information through informative prior distributions and to provide exact inferences for incidence and intervention effects — the information most relevant to policy makers and public health officials.

Through our study, we estimated the epidemic effective population size (the overall number of individuals at immediate risk of infection) to be around 16,000. This number is much smaller than the demography-based estimate of the susceptible population that one would usually consider in a standard epidemic model. Indeed, the combined population of North Kivu, South Kivu, and Ituri provinces exceeds 16 million and accounts for approximately 15% of the DRC population, with many large population centers (e.g., Goma) exceeding half a million inhabitants. This discrepancy between demographic estimates and the estimated effective size of the susceptible population emphasizes the individual-based nature of our analysis and reflects the effects of public health intervention efforts (in particular, ring vaccination and contact tracing) that largely prevented the wide and uncontrolled community spread of the EVD.

Our analysis also indicated that, in different epidemic waves, the average removal time was statistically different with the shortening of the removal time from wave 1 to wave 3 by an average of 1.4 days (from 6 days in wave 1 to 4.6 days in wave 3). This finding is consistent with the general view that increased isolation and vaccination efforts in late 2019 largely contributed to breaking local chains of transmission and ultimately ending the epidemic by mid-2020. Assuming similar infectivity in future outbreaks, our results suggest that, in order to limit the spread of EVD in future outbreaks, a rate of removal similar to that achieved in wave 3 will likely be required.

Although the DRC has successfully contained Ebola outbreaks in the past^6,29^ and an effective vaccine is now available, the security and political challenges in the northern DRC — especially North Kivu and Ituru provinces — continued to create problems for effective public health interventions during the 2018–2020 outbreak. As political challenges in the DRC are likely to persist in the near future, there is great need for a flexible approach in responding to future outbreaks that combines multiple pharmaceutical and non-pharmaceutical strategies. The individual-level EVD data from the 2018–2020 outbreak presented here is, to our knowledge, the first opportunity to comprehensively look at the multi-wave outbreak data and quantitatively assess the strength of non-pharmaceutical interventions while also accounting for the the effects of ring vaccination in decreasing the size of the population at risk of infection. The methodology developed and used here is also of possible relevance for analyzing other outbreaks exhibiting complicated dynamics and multiple incidence waves, including the current COVID-19 pandemic.

## Supplementary Information

Supplementary Information.

## Data Availability

Deidentified dataset and the code used in MCMC analysis are publicly available via Zenodo platform at https://doi.org/10.5281/zenodo.6104188. Further data may be available upon a reasonable request to the authors.
