# Supplementary material for: Analysis of individual-level data from 2018–2020 Ebola outbreak in Democratic Republic of the Congo
Source: Sci Rep. 2022 Apr 1;12:5534. doi: 10.1038/s41598-022-09564-4 (PMC8972744; doi:10.1038/s41598-022-09564-4)
Supplement: Supplementary file 1 — Supplementary Information. [file 41598_2022_9564_MOESM1_ESM.pdf]

Supplementary Material:
Analysis of Individual-level Data from 2018-2020 Ebola Outbreak in Democratic Republic of the Congo

Harley Vossler[1], Pierre Akilimali[2], Yuhan Pan[1], Wasiur R. KhudaBukhsh[3], Eben Kenah[1], and Grzegorz A. Rempała[1,*]

[1]*College of Public Health, The Ohio State University*
[2]*College of Public Health, University of Kinshasa*
[3]*School of Mathematical Sciences, University of Nottingham*

March 2, 2022

A Additional Figures

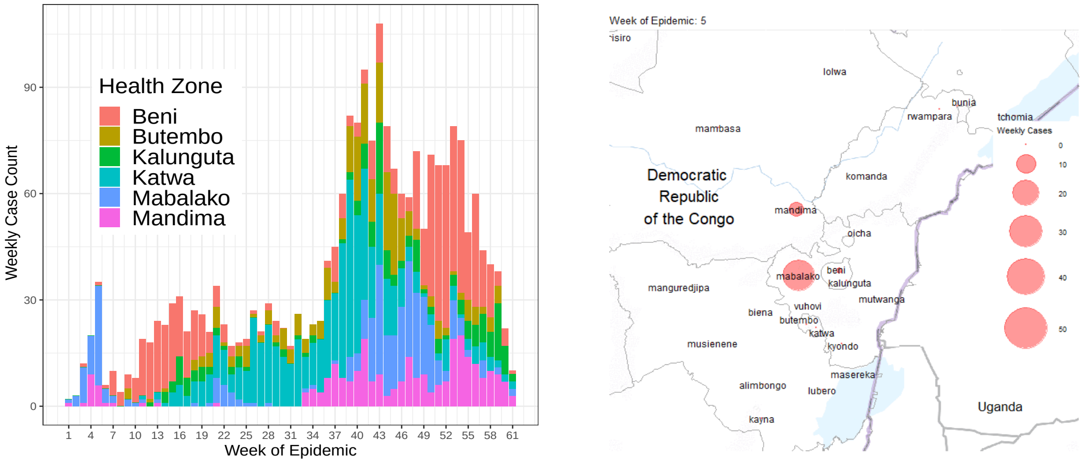

Figure A.1: **Weekly Case Count in DRC**. Left: Proportion of cases, aggregated by week, for each of the six most outbreak-affected health zones: Beni, Butembo, Kalunguta, Katwa, Mabalako, Mandima (top to bottom in legend). Right: temporal and spatial distribution of the DRC cases. For full animation, see `https://github.com/wasiur/DSAofEbola/blob/main/drc_animate_final5.gif`. The animated map was created using open software R [17] with geospatial data obtained from [18].

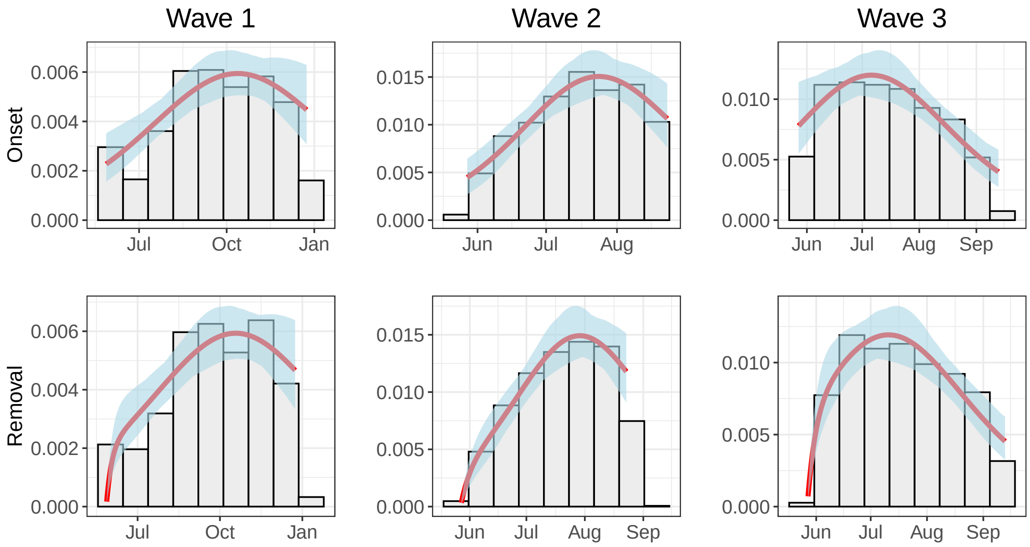

Figure A.2: **Daily Incidence and Removal Rates** The model fit versus actual daily data on cases incidence (onset) and removal. As described in Section 2.3, the analysis was conducted for each wave separately, leading to different sets of parameter values. To facilitate the comparison with Figure 3, the results are plotted as relative counts (density functions).

B Derivations for DSA Model

The idea of dynamic survival analysis (DSA) is to derive individual-level independent Markov processes such that their aggregation leads to the desired ecological models, see [10] and also more recently [31, 32]. In case of the SIR model, equation (1) describes for each individual member of the population their probabilities of being in the susceptible (S), infected (I), or recovered (R) state. This can be done by interpreting the rescaled proportions s_t, ι_t, and r_t as the respective state probabilities whose evolution is described by the stochastic *master equation* [25] given by equation (1) with a random initial condition.

As pointed out in [10], an alternative way of deriving the DSA-based individual infection model from (1) follows from the so-called *Sellke construction* (see, e.g., [33] chapter 2.2) that can be viewed as an agent-based description of the classical, Markovian SIR model of an epidemic. According to the construction, in the population of m initially susceptible individuals, with additional I_0 initially infected, the probability that a randomly selected initially susceptible individual remains susceptible at time $t > 0$ is given by the expression

$$S_t = e^{-\frac{\beta}{m}\int_0^t I_u\, du} \tag{B.1}$$

where I_u is the total number of infected at time u. For the Markovian model, i.e., when infectious period is exponentially distributed with rate parameter γ, the quantity I_u/m converges in probability to the deterministic function ι_u which satisfies (1) (see, e.g., [33] chapter 5.3). Therefore, as the approximation of (B.1) we may take

$$s_t = e^{-\beta\int_0^t \iota_u du} = e^{-\mathcal{R}_0 r_t} \tag{B.2}$$

where (s_t, ι_t, r_t) is the solution of (1). As shown in [10] it follows from the law of large numbers for Poisson

processes that s_t converges to S_t as $m \to \infty$ uniformly in probability over any finite interval $[0, T]$. The DSA interpretation of the SIR model leads to the derivation of the individual-level likelihood function as follows.

Likelihood. We assume that observations of new infections and recoveries are available up to some time horizon T such that $T \in [0, \infty]$. Denote

$$\tau_T = 1 - s_T. \tag{B.3}$$

Note that τ_T is non-decreasing in T and that $\tau_\infty = \tau < 1$, the final epidemic size. In view of (B.2) the quantity $(s_t + \tau_T - 1)/\tau_T$ may be readily interpreted as the statistical survival function [30] on $[0, T]$. It follows that $-\dot{s}_t/\tau_T$ may be interpreted as a density function of infection times on $[0, T]$ for any $T \leq \infty$. Recalling that under the DSA Markov assumption the infectious period is exponentially distributed with rate parameter γ we obtain the following individual-level likelihood function for an initially susceptible individual i observed until time T with infection and recovery times t_i and T_i, respectively:

$$\mathcal{L}(\theta | t_i, T_i, T) = (-\tau_T)^{-1} \dot{s}_{t_i} \gamma^{w_i} e^{-\gamma(T_i \wedge T - t_i)}. \tag{B.4}$$

Here, w_i is the event indicator satisfying $w_i = 0$ if $T_i \wedge T = T$ and $w_i = 1$ otherwise. The likelihood for the set of n individuals in the population with complete records (3) is simply the product of the individual likelihoods, reflecting the assumption that the infection events are approximately independent in a large population.

Likelihood with missing data. As discussed in the main body of the paper, in about 30% of the DRC Ebola cases the individual disease records were incomplete, missing either infection (t_i) or recovery (T_i) times. Fortunately, such missingness may be handled by the DSA likelihood without any need for data imputation. In case when only t_i is observed, (T_i is missing), the likelihood (B.4) reduces simply to

$$\mathcal{L}(\theta | t_i, \circ, T) = (-\tau_T)^{-1} \dot{s}_{t_i}. \tag{B.5}$$

On the other hand, if only T_i is observed (t_i is missing), the likelihood is obtained from the convolution formula and (1)

$$\mathcal{L}(\theta | \circ, T_i, T) = (-\tau_T)^{-1} \int_0^{T_i} \dot{s}_u \gamma e^{-\gamma(T_i - u)} du = \frac{\gamma}{\tau_T} (\iota_{T_i} - \rho e^{-\gamma T_i}). \tag{B.6}$$

Similarly as above, the likelihood for incomplete data (4) is obtained by taking product of (B.5) and (B.6) over all individual incomplete histories.

Effective population size and outbreak size. Since only infections and recoveries are recorded are recorded during a typical epidemic, it is often difficult to determine the size (N) of the susceptible population at risk of infection. This is known in the literature as the problem of estimating N the *effective population size* [10]. Under the DSA interpretation of the SIR model, this estimate may be obtained as

$$\hat{N} = \frac{k_T}{\tau_T}, \tag{B.7}$$

where k_T is the count of observed infected over the time horizon T and τ_T is given by (B.3). Similarly, one may also estimate the final epidemic count K_∞ of all already observed and future infections by

$$\hat{K}_\infty = \hat{N}\tau, \tag{B.8}$$

where $\tau = \lim_{T\to\infty} \tau_T$ is the final epidemic size.

References

[31] Ido Somekh, Wasiur R. KhudaBukhsh, Elisabeth Dowling Root, Grzegorz A Rempała, Eric Simões, and Eli Somekh. Quantifying the population-level effect of covid-19 mass vaccination campaign in Israel: A modeling study. *Open Forum Infectious Diseases*, 2022. ofac087 `https://doi.org/10.1093/ofid/ofac087`.

[32] Wasiur R. KhudaBukhsh, Sat Kartar Khalsa, Eben Kenah, Grzegorz A. Rempala, and Joseph H. Tien. COVID-19 dynamics in an Ohio prison. *medRxiv*, 2021.

[33] Hakan Andersson and Tom Britton. *Stochastic epidemic models and their statistical analysis*, volume 151. Springer Science & Business Media, 2012.
